# Functional characterization of the Arabidopsis transcription factor bZIP29 reveals its role in leaf and root development

**DOI:** 10.1093/jxb/erw347

**Published:** 2016-09-22

**Authors:** Jelle Van Leene, Jonas Blomme, Shubhada R Kulkarni, Bernard Cannoot, Nancy De Winne, Dominique Eeckhout, Geert Persiau, Eveline Van De Slijke, Leen Vercruysse, Robin Vanden Bossche, Ken S Heyndrickx, Steffen Vanneste, Alain Goossens, Kris Gevaert, Klaas Vandepoele, Nathalie Gonzalez, Dirk Inzé, Geert De Jaeger

**Affiliations:** ^1^Department of Plant Systems Biology, VIB, B-9052 Gent, Belgium; ^2^Department of Plant Biotechnology and Bioinformatics, Ghent University, B-9052 Gent, Belgium; ^3^Department of Medical Protein Research, VIB, B-9000 Gent, Belgium; ^4^Department of Biochemistry, Ghent University, B-9000 Gent, Belgium

**Keywords:** *Arabidopsis thaliana*, bZIP group I transcription factors, cell proliferation, cell wall, chromatin immunoprecipitation, leaf cell number, plant development, root cell number, tandem affinity purification.

## Abstract

Plant bZIP group I transcription factors have been reported mainly for their role during vascular development and osmosensory responses. Interestingly, bZIP29 has been identified in a cell cycle interactome, indicating additional functions of bZIP29 in plant development. Here, bZIP29 was functionally characterized to study its role during plant development. It is not present in vascular tissue but is specifically expressed in proliferative tissues. Genome-wide mapping of bZIP29 target genes confirmed its role in stress and osmosensory responses, but also identified specific binding to several core cell cycle genes and to genes involved in cell wall organization. bZIP29 protein complex analyses validated interaction with other bZIP group I members and provided insight into regulatory mechanisms acting on bZIP dimers. In agreement with *bZIP29* expression in proliferative tissues and with its binding to promoters of cell cycle regulators, dominant-negative repression of bZIP29 altered the cell number in leaves and in the root meristem. A transcriptome analysis on the root meristem, however, indicated that bZIP29 might regulate cell number through control of cell wall organization. Finally, ectopic dominant-negative repression of bZIP29 and redundant factors led to a seedling-lethal phenotype, pointing to essential roles for bZIP group I factors early in plant development.

## Highlight

bZIP29, an Arabidopsis transcription factor, is expressed in proliferative tissues and involved in the regulation of cell number in the root meristem and leaves.

## Introduction

Transcription factors (TFs) represent one of the most extended functional protein classes in eukaryotes. In the model plant *Arabidopsis thaliana* (Arabidopsis), TFs account for 9% of the protein-encoding gene pool (Pruneda-Paz *et al.*, 2014). TFs are mostly classified according to their DNA-binding domains. One of the largest TF families in the eukaryotic kingdom is characterized by the presence of a basic region–leucine zipper (bZIP) domain. The basic region binds DNA, while the leucine zipper motif is required for dimerization. During plant evolution, this family of TFs has expanded greatly. Arabidopsis, for example, possesses up to four times more bZIP TF members compared with yeast, worm and human (Riechmann *et al.*, 2000). In Arabidopsis, 75 bZIP TFs have been identified, which were clustered into subgroups based on sequence similarity in their basic region and the presence of additional conserved motifs (Jakoby *et al.*, 2002). According to additional screens, however, up to 82 bZIP TFs are present in Arabidopsis (Qu and Zhu, 2006), being involved in a plethora of biological processes (Alves *et al.*, 2013; Llorca *et al.*, 2014).

One bZIP clade that is less well characterized is group I, containing 13 members in Arabidopsis, and sharing a lysine residue in the basic domain that replaces the highly conserved arginine residue (Jakoby *et al.*, 2002). Several orthologous plant bZIP group I proteins have been studied, mainly indicating a role in vascular development (Torres-Schumann *et al.*, 1996; Yin *et al.*, 1997; Ringli and Keller, 1998; Fukazawa *et al.*, 2000; Petruccelli *et al.*, 2001; Dai *et al.*, 2003; Dai *et al.*, 2004). Likewise, in Arabidopsis, the bZIP group I subfamily is expressed predominantly in vascular tissues (Pyo *et al.*, 2006). The best-studied group I member in Arabidopsis is VirE2-interacting protein 1 (VIP1; bZIP51). This multifunctional protein has initially been identified for its role in the Agrobacterium response (Tzfira *et al.*, 2001, 2002; Ward *et al.*, 2002; Li *et al.*, 2005), and has been shown to play a role in pathogen response (Djamei *et al.*, 2007; Pitzschke *et al.*, 2009), oxidative stress, and heavy metal and low-sulphur responses (Wu *et al.*, 2010). In vascular tissues, VIP1 and interacting redundant bZIP group I members regulate osmosensory signaling (Tsugama *et al.*, 2012, 2014).

There is evidence that VIP1 acts not only in (a)biotic stress responses, but also in cell proliferation and plant development. VIP1 has been implicated in regulation of suppression of differentiation and shoot formation (Avivi *et al.*, 2004). Moreover, its activity is raised in dividing cells (Lacroix and Citovsky, 2013) and *VIP1* overexpression leads to an increased leaf cell number and decreased cell size (Blomme *et al.*, 2014).

Interestingly, also bZIP29 has been linked to cell proliferation because it was identified as an interactor of different cell cycle regulators (Van Leene *et al.*, 2010). Therefore, we further investigated its role in plant development. First of all, bZIP29 was specifically expressed in proliferating tissues. Through chromatin immunoprecipitation, target genes of bZIP29 were identified, not only validating binding of bZIP group I members to stress or osmosensory genes, but in addition showing binding to cell cycle regulators and multiple genes involved in cell wall organization. Protein complex analyses uncovered the bZIP29 heterodimerization landscape, and provided insight into regulatory mechanisms acting on bZIP group I dimers. Dominant-negative repression of bZIP dimers comprising bZIP29 not only revealed that bZIP29 controls cell number, both in the root meristem and in the leaves, but also that this subfamily is essential early in plant development.

## Materials and methods

### Generation of transgenic lines and plant growth conditions

The T-DNA insertion line of *bZIP29* (GABI1211B01; T-DNA in first exon) was obtained from the GABI-KAT collection and genotyping (Supplementary Table S1 at *JXB* online) confirmed that the line was homozygous for the insert. The SRDX domain was C-terminally fused to the CDS of bZIP29 by PCR (Supplementary Table S1). An entry vector with bZIP29 fused to the SRDX domain was obtained by a Gateway BP reaction (Invitrogen/Thermo Fisher Scientific). For overexpression (P35S) of *bZIP29* or the *bZIP29-SRDX* fusion, the entry vectors were recombined with the pFAST-G02 vector (Shimada *et al.*, 2010) by Gateway LR reactions (Invitrogen/Thermo Fisher Scientific). For expression of the *bZIP29-SRDX* fusion under control of the endogenous promoter, a 2-kb promoter fragment was amplified by PCR (Supplementary Table S1) using Phusion High-Fidelity DNA polymerase (Thermo Fisher Scientific) following the manufacturer’s protocol, and cloned into pDONRP4P1R. The resulting vector was used in a Gateway LR reaction with the bZIP29-SRDX entry vector, pEN-R2-9-L3 (Karimi *et al.*, 2007) and pK8m34GW, in which the FAST cassette was inserted in the backbone. Expression vectors were transformed into *Agrobacterium tumefaciens* (C58C1 Rif^R^ pMP90) for floral dip of Arabidopsis Col-0. Transformed seeds were selected based on fluorescence in the seed coat. Plants were grown *in vitro* in half-strength (½) Murashige and Skoog (MS) medium (Murashige and Skoog, 1962) supplemented with 1% sucrose at 21 °C under a 16h day/8h night regime. Plants grown in soil were exposed to the same day length. For analysis of *Promoter_bZIP29:bZIP29-SRDX* or *P35S:bZIP29* overexpressor lines, homozygous T3 plants were generated harboring one T-DNA insertion. For comparisons with wild-type, out-segregated wild-type plants were generated.

### Green fluorescent protein–β-glucuronidase-based expression analysis

For green fluorescent protein (GFP)–β-glucuronidase (GUS)-based expression analysis, entry vectors containing 2-kb promoter fragments were recombined into the *NLS-GFPGUS* vector pMK7S*NFm14GW (Karimi *et al.*, 2007) by Gateway LR reactions and the expression vector was transferred to Arabidopsis. Tissues of multiple developmental stages were analysed by GUS staining with an Olympus microscope (DIC-BX51) (Grunewald *et al.*, 2008) or by confocal microscopy with a Zeiss LSM 510. Roots were incubated in propidium iodide (3mg L^–1^), and washed with and subsequently mounted in Milli-Q H_2_O.

### Tandem affinity purification and mass spectrometry analysis

For tandem affinity purification (TAP) in cell culture, cloning, cell culture transformation, tandem affinity purification and mass spectrometry were performed as described (Van Leene *et al.*, 2011).

For TAP on seedlings, C-terminal GSrhino (Van Leene *et al.*, 2015) TAP fusion vectors were generated using the moderate promoter of a cyclin-dependent kinase (CDK) of type A, CDKA;1 (Eloy *et al.*, 2012). Biomass generation, TAP and mass spectrometry were performed as described (Van Leene *et al.*, 2015), and a list of non-specific proteins was subtracted, except if semi-quantitative analyses identified proteins as specific (Van Leene *et al.*, 2015). Protein identification details are listed in Supplementary Table S2.

### Tandem chromatin affinity purification sequencing analysis

For tandem chromatin affinity purification sequencing (TChAP-seq) analyses, the *P35S:HBH-bZIP29* vector was constructed by a Gateway LR reaction in the pKNHBH vector. Transgene cell cultures were generated (Van Leene *et al.*, 2011) and TChAP was performed as described (Verkest *et al.*, 2014). *P35S:HBH-bZIP29* (in duplicate) and mock wild-type PSB-D TChAP DNA libraries were prepared and sequenced on a Genome II Analyzer (Illumina). Quality control and mapping of reads was performed as described (Vercruyssen *et al.*, 2014). Peak calling with uniquely mapped reads was done against the mock control using MACS (Zhang *et al.*, 2008) (version 2.0.10.2; MFOLD parameters [5, 30]; effective genome size 100Mb). Peak annotation was done using peak summits against the TAIR10 gene annotation. Peaks in common for both replicates were determined using intersectBed (Quinlan and Hall, 2010), with at least 25% peak overlap. For *de novo* motif finding, peak-motifs from RSATools was used with default settings (Thomas-Chollier *et al.*, 2012). Enrichment of motifs was calculated by generating 1000 random control samples from the genome and the *p*-value of the enrichment was defined as the number of times the motif occurred as frequently in the control regions as in the real dataset, over the 1000 samples. The similarity of *de novo* motifs with known motifs was determined using compare-matrices from RSATools with lower threshold limit 0.5, against a collection of known motifs (Higo *et al.*, 1999; Davuluri *et al.*, 2003; Steffens *et al.*, 2004; Weirauch *et al.*, 2014). The bZIP29 TChAP-seq data have been deposited in the ArrayExpress database (http://www.ebi.ac.uk/arrayexpress) under accession number E-MTAB-3754.

### GAL4 transactivation and transient expression assay

For GAL4 transactivation assays, N-terminal GAL4 fusions under control of the 35S promoter (*P35S:GAL4-bZIP29*, *P35S:GAL4-bZIP29-SRDX*) were generated by Gateway LR reactions in pm43GW7. For transient expression assays, the *XTH9* promoter was cloned in pWGL7 and co-transfected with *p2GW7* overexpression effector constructs (*bZIP29* and/or *VIP1*). GAL4 transactivation and transient expression assays in tobacco Bright Yellow-2 (BY-2) protoplasts were performed as described (Vanden Bossche *et al.*, 2013; Gonzalez *et al.*, 2015). Student’s *t*-test was used to confirm statistically significant differences between control and effector constructs (*P*<0.001).

### Leaf growth analysis

Leaf series analysis, leaf cell number and cell size determination, and dental resin imprints were done as described (Gonzalez *et al.*, 2015), on plants grown under *in vitro* conditions, at 21 days after stratification (DAS).

### Root growth analysis

Primary root length and lateral root density were determined 10 DAS. To score root growth defects, plants were grown for 7 DAS on ½MS plates inclined at an angle of approximately 45°. For gravistimulation experiments, roots of 4-day-old light-grown seedlings, germinated on vertical plates, were aligned, reoriented by a 90° angle and placed in the dark. Plates were scanned after 6h and 20–24h and the root bending angle was determined using ImageJ software. For root meristem length determination, plants were grown vertical for 5 DAS. After propidium iodide staining, root meristem length was measured from the quiescent center (QC) until the first cortex cell that elongates.

### RNA-seq transcriptome analysis and quantitative PCR confirmation

For RNA-seq, *Promoter_bZIP29:bZIP29-SRDX* line 1 and out-segregated WT line 1 plants were grown for 5 DAS on nylon meshes (Prosep), placed on vertical ½MS medium-containing plates. In three biological repeat experiments, root tips (<3mm) were harvested. Total RNA was isolated using the RNeasy Plant Mini Kit (Qiagen) and treated with RQ1 RNase-Free DNase I (Promega). RNA samples were processed by preparing a Truseq RNA-seq library (Illumina) and sequenced on an Illumina HiSeq 2000 (50 nt single reads) at GATC Biotech Ltd (Cologne, Germany). The quality of the raw data was verified with FASTQC (http://www.bioinformatics.babraham.ac.uk/projects/fastqc/). Sequences were filtered and trimmed using FASTX-Toolkit (http://hannonlab.cshl.edu/fastx_toolkit/; version 0.0.13). Reads were mapped to the Arabidopsis reference genome (TAIR10) using GSNAP version 2012-07-20 (Wu and Nacu, 2010). The uniquely mapping reads were used for quantification on the gene level with htseq-count from the HTSeq python package (Anders *et al.*, 2015). These analyses were performed using a local Galaxy instance (http://galaxyproject.org) (Giardine *et al.*, 2005). Data were normalized with trimmed mean of M-values, implemented in edgeR (Robinson *et al.*, 2010). Differentially expressed genes were defined by a twofold difference between samples with a *P*-value <0.05 at false discovery rate (FDR) <0.05. The significance of the overlap between the RNA-seq down-regulated dataset and the TChAP-seq dataset was calculated using the hypergeometric distribution function. The RNA-seq data discussed in this article have been deposited in the ArrayExpress database (http://www.ebi.ac.uk/arrayexpress) under accession number E-MTAB-3755.

For quantitative PCR (qPCR) confirmation of identified targets, RNA was isolated from the root tips prior to cDNA synthesis with the iScript cDNA Synthesis Kit (Bio-Rad). Relative expression levels were determined by qRT-PCR with the LightCycler 480 Real-Time SYBR Green PCR System (Roche). Two reference genes were used for normalization (ACTINE_2-2 AT3G18780 and AteEF1A-4 AT5G60390). For qPCR analysis of *SRDX* levels in the three *Promoter_bZIP29:bZIP29-SRDX* lines, RNA was isolated from seedlings at 21 DAS. Normalization was obtained with *PP2A* (AT1G13320) as reference gene.

## Results

### A detailed view on the expression profile of bZIP29

**Fig. 1.**
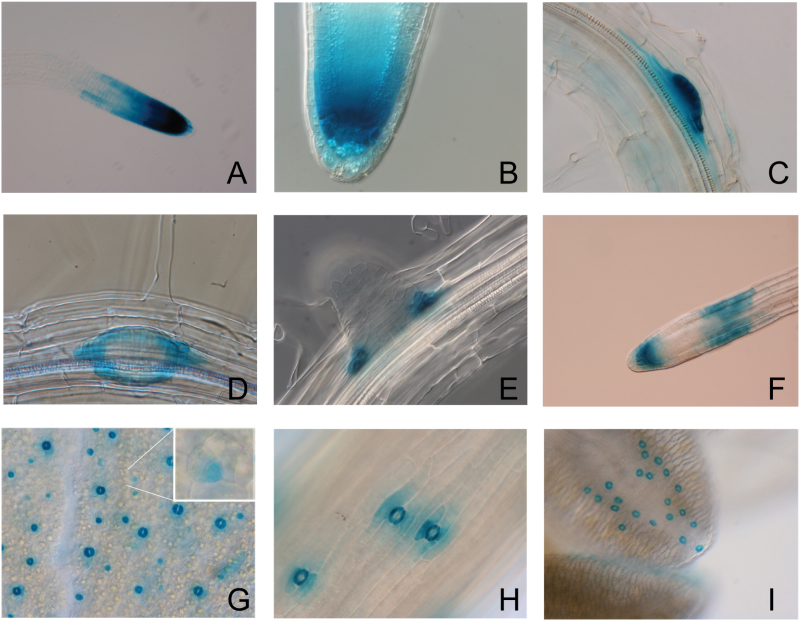
*bZIP29* expression analysis. (A–I) Histochemical GUS staining of a *Promoter_bZIP29:GFP-GUS* reporter line. In roots, expression was detected in the primary root tip (A) with maximum expression in the QC and the columella cells (B), in lateral root primordia (C) with maximum expression at the boundaries (D and E), and later during lateral root development in the lateral root tip and at the transition from the proliferation to expansion zone (F). In leaves, strong expression was detected in fully developed guard cells (G), and in the progenitor meristemoid (G, inset). This stomatal expression pattern was confirmed in the stomata present in the hypocotyl (H) and in the anthers (I). (This figure is available in color at *JXB* online.)

In addition to their proposed function in vascular development, the bZIP group I subfamily might have additional roles, because some members (bZIP29, VIP1, bZIP30, bZIP52, and bZIP59/PosF21) appear to be expressed also in the root meristem (Pyo *et al.*, 2006). To investigate the spatio-temporal expression pattern of *bZIP29* during plant development, 2kb upstream of the start codon was used as the promoter fragment to drive the expression of a green fluorescent protein (GFP)–β-glucuronidase (GUS) reporter. Interestingly, *bZIP29* showed very specific expression patterns (Fig. 1), mainly restricted to proliferative tissues, while no expression was detected in vascular tissues. *bZIP29* was highly expressed in the root meristem of the primary root (Fig. 1A). Shorter GUS staining allowed us to restrict the expression region to the distal part of the root meristem, with maximum expression observed in the quiescent center (QC) and the columella cells (Fig. 1B). Later, *bZIP29* was expressed in lateral root primordia (Fig. 1C), more specifically at the boundaries of the lateral root (Fig. 1D, E) and in the lateral root meristem (Fig. 1F). Furthermore, *bZIP29* expression was activated during lateral root development, at the transition of the proliferation to expansion zone (Fig. 1F). In leaves, *bZIP29* was expressed in meristemoids, guard mother cells and very strongly in stomata (Fig. 1G). This stomatal expression was also confirmed in the guard cells present in hypocotyls (Fig. 1H) and in anthers (Fig. 1I). In summary, *bZIP29* is expressed in proliferative tissues and not in vascular tissues, confirming earlier observations (Pyo *et al.*, 2006), and pointing to additional functions for members of this subfamily next to their role in vascular development.

### Genome-wide discovery of genes bound by bZIP29

**Fig. 2.**
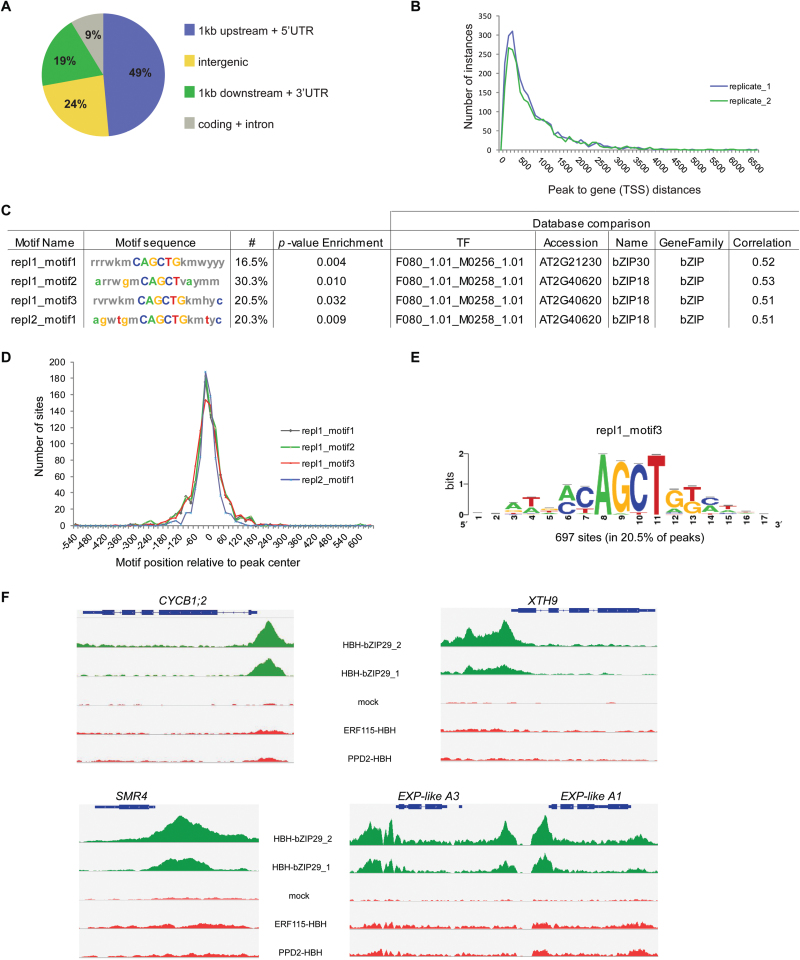
Genome-wide identification of genes bound by bZIP29 through TChAP-seq analysis. (A) Genome-wide distribution of bZIP29 TChAP-seq peaks present in both replicates, in relation to the gene structure. (B) Distance of the summit of the peaks from both replicates, relative to the translation start site (TSS) of the nearest gene. (C) *De novo* motif analysis of the TChAP-seq data, identifying significant over-representation of similar motifs in both replicates. Through database comparison, similarity to motifs bound by bZIP30 and bZIP18 was found. (D) Sequence position location (bp) of the identified motifs in relation to the peak summit. (E) Logo representing motif 3 identified in the first replicate. (F) Integrative Genomics Viewer (IGV) screenshot (Thorvaldsdóttir *et al.*, 2013) of sequenced reads from bZIP29, mock, ERF115 and PPD2 TChAP-seq experiments, mapped to the mitotic cell cycle regulator *cyclin B1;2*, the cell cycle inhibitor *SMR4*, and the cell wall organizing genes *XTH9*, *EXP-like A1* and *EXP-like A3*. The gene regions are represented by bars. (This figure is available in color at *JXB* online.)

To identify target genes bound by bZIP29, tandem chromatin affinity purification (TChAP) experiments were performed in duplicate. After correction against the negative control, 1678 peaks were found in common in both replicates (Supplementary Table S3), representing binding to 1564 genes. The binding landscape of bZIP29 was specific, because 1064 genes were bound by bZIP29 and not by ERF115 (Heyman *et al.*, 2013) nor by Peapod2 (Gonzalez *et al.*, 2015) (Supplementary Table S3), two TFs that were analysed with the TChAP procedure in the same next-generation sequencing run. As expected for a TF, the majority of the binding events took place in the 1-kb upstream, 5′UTR and intergenic regions (Fig. 2A), and most of the peaks had their summit located in the 1kb upstream of the translation start site (Fig. 2B).

*Cis*-regulatory elements targeted by bZIP29 were identified through a *de novo* motif discovery analysis. Both replicates revealed significant enrichment of similar motifs (Fig. 2C), located preferentially near the peak summit (Fig. 2D). Through comparison with a database of known motifs, high sequence similarity was observed with motifs linked to bZIP group I members bZIP30 and bZIP18 (Weirauch *et al.*, 2014) (Fig. 2C). The core of these motifs (mCAGCTGk) (Fig. 2E) resembles the known VIP1 response element (VRE), which is responsible for activation of the stress genes encoding MYB44 and thioredoxin Trxh8 (Pitzschke *et al.*, 2009). In addition, it has been shown that VIP1 and bZIP29 have partially redundant functions in osmosensory response (Tsugama *et al.*, 2012, 2014) through binding to a hypo-osmolarity-responsive element (AGCTGk) in the promoter of the hypo-osmolarity-responsive genes *CYP707A1* and *CYP707A3*, encoding two key enzymes involved in abscisic acid (ABA) degradation. All these four known VIP1 targets were validated in the bZIP29 target list (Supplementary Fig. S1). Moreover, of the 26 tested rehydration- or submergence-responsive genes containing the hypo-osmolarity-responsive element in their promoter (Tsugama *et al.*, 2014), 15 were also bound by bZIP29, and three of them were among the 20 most significantly bound genes of bZIP29 (Supplementary Table S3). These results indicate that bZIP29 and VIP1 have similar functions in osmosensory signaling and stress responses. Furthermore, the TChAP data show that bZIP group I factors bZIP29, bZIP30, bZIP18 and VIP1 target similar *cis*-regulatory elements.

To gain more insight into genes controlled by bZIP29, a gene ontology enrichment analysis using the PLAZA platform (version 3.0 Dicots) (Proost *et al.*, 2015) was performed on the genes bound by bZIP29 (Supplementary Table S4). In line with the redundant functions of bZIP29 and VIP1, hyperosmotic response and response to stress were among the enriched biological processes. Furthermore, enrichment was observed for cell growth, developmental growth, meristem growth, meristem maintenance, and regulation of cyclin-dependent protein serine/threonine kinase activity. Moreover, almost 9% of the bZIP29 targets are periodically expressed during cell proliferation (Van Leene *et al.*, 2010), representing a highly significant over-representation of cell cycle-regulated genes (*P*-value <0.001) (Supplementary Table S3). Interestingly, the mitotic B-type cyclin *CYCB1;2* was identified as one of the most significantly bound genes, and the binding in the *CYCB1;2* promoter region was specific for bZIP29 (Fig. 2F). Additional cell cycle regulators, such as the cell cycle inhibitor Siamese-related 4 (*SMR4*), were specifically targeted by bZIP29 (Fig. 2F). Analysis of the cellular compartments that are over-represented in the gene list bound by bZIP29 revealed high enrichment for proteins localized in the cell wall (Supplementary Table S4). One of the most over-represented molecular functions was xyloglucan:xyloglucosyl transferase activity (Supplementary Table S4), indicating that bZIP29 regulates genes involved in rearrangements of the cell wall. Xyloglucan endotransglucosylase (*XTH9*) for example was specifically bound by bZIP29 (Fig. 2F). This protein functions in loosening and rearrangement of the cell wall (Hyodo *et al.*, 2003) and is specifically expressed in the QC (Nawy *et al.*, 2005). Two other bZIP29 targets with a role in cell wall organization were two neighboring genes encoding expansin-like proteins (Fig. 2F). These results indicate that bZIP29 might regulate cell wall organization in proliferating cells.

### Comprehensive analysis of the bZIP29 protein interactome

Previously, yeast two-hybrid (Y2H) and bimolecular fluorescence complementation (BiFC) assays demonstrated that several bZIP group I members, such as VIP1, not only form homodimers but also heterodimers with other bZIP group I members (Tsugama *et al.*, 2014).

To obtain a more *in vivo* comprehensive view on the protein complexes in which bZIP29 functions, TAP experiments were performed from both Arabidopsis seedlings and cell cultures. The TAP analyses confirmed that bZIP29 interacts mainly with members of the group I subfamily (Table 1). The interaction of bZIP29 with VIP1, bZIP30, bZIP52, and bZIP59/PosF21 might occur in the root meristem, because all members are co-expressed in this tissue (Pyo *et al.*, 2006). To analyse in which tissue bZIP29 could interact with bZIP69, we analysed *bZIP69* expression (Supplementary Fig. S2), showing overlapping expression profiles in lateral root primordia, where they might function as a heterodimer. The expression analysis confirmed that *bZIP69* is not expressed in the root meristem and not in vascular tissues (Pyo *et al.*, 2006). In addition to the lateral root primordia, *bZIP69* expression was observed in the lateral root cap, in the shoot apical meristem, in hydathode pores, and during flower development.

**Table 1.** *Identification of bZIP29 interactors by tandem affinity purification on Arabidopsis cell culture and seedling extracts* Proteins identified by TAP with bZIP29 as bait proteins are listed, either found in cell cultures or in seedlings. Only proteins identified with at least two significant peptides, of which one was unique, are retained. C-GS: C-terminal TAP fusion to GS TAP tag; N-GS: N-terminal TAP fusion to GS TAP tag. Identification of a prey protein over the different TAP experiments was counted.

Prey			Cell culture		Seedlings
Locus	Name	Type	C-GS (2)^*a*^	N-GS (4)	C-GS (2)
AT4G38900	AtbZIP29	bZIP class I	2	4	2
AT1G06070	AtbZIP69	bZIP class I	1	4	2
AT1G43700	AtbZIP51 VIP1	bZIP class I	2	3	2
AT2G31370	AtbZIP59 PosF21	bZIP class I	1	2	2
AT2G40620	AtbZIP18	bZIP class I	2	3	2
AT2G21230	AtbZIP30	bZIP class I		1	2
AT1G06850	AtbZIP52	bZIP class I			2
AT3G58120	AtbZIP61	bZIP class E			2
AT2G42380	AtbZIP34	bZIP class E			1
AT1G58110	bZIP TF family protein	Unclassified bZIP			2
AT4G09000	GRF1 GF14 chi	14-3-3	2	4	2
AT3G02520	GRF7 GF14 nu	14-3-3	2	3	
AT5G38480	GRF3 GF14 psi	14-3-3	2	3	2
AT1G78300	GRF2 GF14 omega	14-3-3	2	1	1
AT5G65430	GRF8 GF14 kappa	14-3-3	2	2	
AT1G22300	GRF10 GF14 epsilon	14-3-3		3	1
AT5G10450	GRF6 GF14 lambda	14-3-3		2	2
AT1G35160	GRF4 GF14 phi	14-3-3		2	
AT2G42590	GRF9 GF14 mu	14-3-3	2	1	
AT5G28850^*b*^	ATB′′ε	Phosphatase	1	2	1
AT5G44090	ATB′′α	Phosphatase	1	2	
AT2G42500^*b*^	PP2A-4	Phosphatase	2		

^*a*^Total number of experiments per bait fusion is shown in parentheses.

^*b*^The two phosphatase subunits AT5G28850 and AT2G42500 might be encoded respectively by AT5G28900 (ATB′′δ) and AT3G58500 (PP2A-3), as these encode isoforms that cannot be discriminated on the protein level by MS.

Besides interactions with group I members, TAP in seedlings revealed interaction of bZIP29 with both group E members (bZIP34 and bZIP61) and with one unclassified bZIP TF (AT1G58110). Group E bZIP factors are highly similar to group I members in their leucine zipper motif (Jakoby *et al.*, 2002), but they do not contain the lysine at position –10 in the basic region that is characteristic for group I members (Supplementary Fig. S3). Group E members possess a proline residue in the third leucine heptad preventing homodimerization, and the TAP data confirmed that group E members form heterodimers with group I members, as previously demonstrated (Shen *et al.*, 2007). Both group E members are highly co-expressed with each other and with two bZIP group I members (bZIP18 and bZIP52) (Obayashi *et al.*, 2009), further supporting the observed interactions.

Moreover, interactions of bZIP29 with 9 out of 13 (DeLille *et al.*, 2001) different 14-3-3 proteins were identified (Table 1). These 14-3-3 general regulatory factors (GRFs) are scaffold proteins known to bind phosphorylated proteins and are mainly involved in signal transduction. Confirmation of interaction between bZIP29 and 14-3-3 proteins was obtained by Y2H (*Arabidopsis* Interactome Mapping Consortium, 2011). *In silico* analysis (Obenauer *et al.*, 2003) discovered two consensus 14-3-3 motifs in bZIP29 (on Ser101 and Ser196), sustaining its direct interaction with 14-3-3 proteins. Finally, bZIP29 co-purified two protein phosphatase 2A (PP2A) regulatory B′′ subunits and one PP2A catalytic subunit (Table 1). According to a co-expression analysis (De Bodt *et al.*, 2012), one of the regulatory subunits (*ATB′′α*) is highly co-expressed with *VIP1* and *bZIP30*, adding extra confidence to the interaction of PP2A complexes with bZIP group I members.

### Generation of dominant-negative repressors for functional analysis

**Fig. 3.**
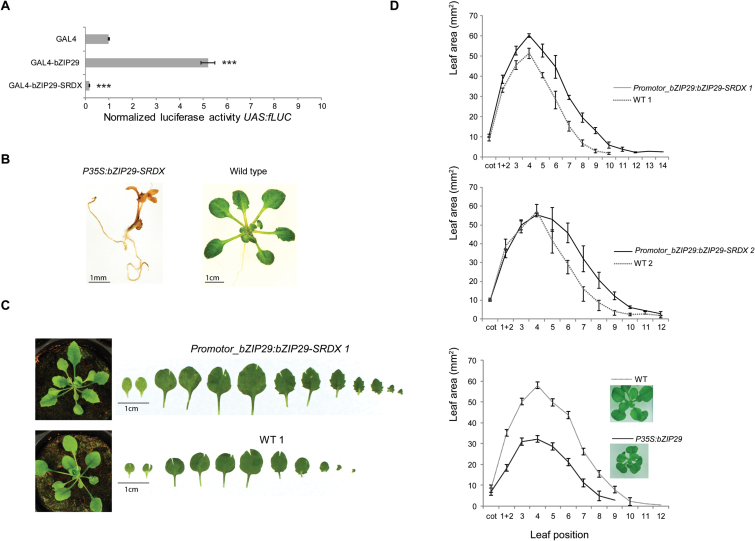
Dominant-negative repression of *bZIP29* and phenotypical analyses of mutant lines. (A) Transactivation activity in tobacco protoplasts transfected with a *UAS:fLUC* reporter construct, or *GAL4DBD* effector constructs fused to *bZIP29* or to *bZIP29-SRDX*. Mean values are compared with the GAL4 control (*n*=8; ***P<0.001, Student’s *t*-test). Fusion to the SRDX motif abolishes the transcriptional capacity of the bZIP29 TF. (B) Seedling-lethal phenotype of the *P35S:bZIP29-SRDX* dominant-negative repressor line at 7 DAS, compared with the wild-type phenotype. (C) Phenotype in soil (28 DAS) and leaf series of *Promoter_bZIP29:bZIP29-SRDX* line 1 and the out-segregated wild-type line 1, at 21 DAS, grown *in vitro*. (D) Area of individual leaves of the two independent *Promoter_bZIP29:bZIP29-SRDX* lines, compared with the corresponding out-segregated wild-type lines, and of the overexpressor line (*P35S:bZIP29*), grown *in vitro*, at 21 DAS (*n*=3). Images show phenotype at 21 DAS, grown *in vitro*. (This figure is available in color at *JXB* online.)

bZIP TFs regulate gene expression through direct binding of their basic DNA-binding domain to target genes (Jakoby *et al.*, 2002) and C-terminal glutamine-rich stretches (Supplementary Fig. S3) stabilize promoter occupancy in concert with the bZIP domain, promoting expression of target genes (Xiao and Jeang, 1998; Mayr *et al.*, 2005). To validate *in planta* that bZIP29 is a transcriptional activator, as was demonstrated in a yeast-one-hybrid assay (Tsugama *et al.*, 2014), a transient expression assay was performed (De Sutter *et al.*, 2005), demonstrating that bZIP29 acts as a transcriptional activator in plant cells (Fig. 3A).

Because overexpression or mutation of *VIP1* did not alter expression of its target gene *CYP707A3* (Tsugama *et al.*, 2012), members of this family must have partially redundant functions. This was validated for bZIP29, because a homozygous T-DNA insertion line of *bZIP29* showed no obvious phenotype when compared with wild-type plants. To cope with redundancy among bZIP group I members, we fused a dominant-negative SRDX repressor domain to the C-terminus of bZIP29 (Hiratsu *et al.*, 2003). A transient GAL4 transactivation assay confirmed that fusion to the SRDX domain abolished the transactivation capacity of bZIP29 (Fig. 3A). Next, the dominant-negative repressor was overexpressed *in planta* from the 35S promoter. Intriguingly, overexpression of the *bZIP29-SRDX* repressor led to a seedling-lethal phenotype (Fig. 3B), which was clearly manifested in 7-day-old seedlings that were very small, pale or brownish and showed a drastic agravitropic response. Dominant-negative repression of *bZIP29* and redundantly interacting bZIP factors thus demonstrates that the group I subfamily is essential early during plant development.

### Leaf phenotypical analysis of a bZIP29 dominant-negative repressor line

To obtain a more precise view on the endogenous function of bZIP29, we expressed the *bZIP29-SRDX* fusion from its endogenous promoter. Homozygous T3 seeds were generated, and two independent lines with slightly varying expression levels of *bZIP29-SRDX* (Supplementary Fig. S4) were selected for phenotypical analyses (Fig. 3C, D). In contrast to overexpression of *bZIP29-SRDX* from the 35S promoter, expression with the endogenous promoter was no longer seedling-lethal. Compared with wild-type, leaves from *Promoter_bZIP29:bZIP29-SRDX* lines were larger and leaf margins were more serrated (Fig. 3C). To obtain a detailed view on the leaf phenotype, the area per leaf was measured (Fig. 3D). For the line with the highest transgene expression level (Fig. 3D, *Promoter_bZIP29:bZIP29-SRDX 1*), the leaf area was significantly increased, although the increase in leaf size was less pronounced for the first two leaves that were fully developed at 21 DAS. In addition, more leaves were formed at that time point. In the transgenic line with a more moderate transgene expression (Fig. 3D, *Promoter_bZIP29:bZIP29-SRDX 2*), we could confirm an increased leaf size for the younger leaves (leaves 5–10), although there was no obvious difference in size for the oldest leaves (leaves 1–4), nor in the amount of leaves.

**Fig. 4.**
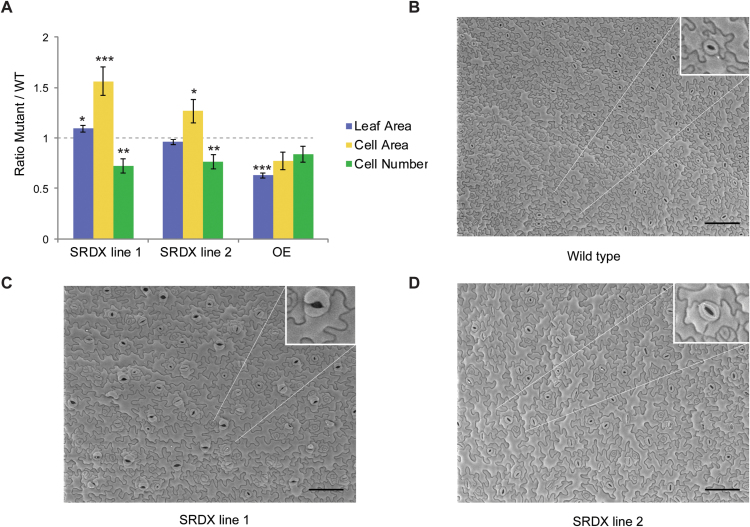
Leaf cellular analysis of *bZIP29* repressor and overexpressor lines. (A) Leaf area, cell area, and cell number of leaf 1 and 2 obtained from two independent *Promoter_bZIP29:bZIP29-SRDX* repressor lines or from the *P35S:bZIP29* overexpressor line, grown *in vitro*, 21 DAS (*n*=5). Normalization was done relative to out-segregated wild-type plants. *P<0.05; **P<0.01; ***P<0.001 (Student’s *t*-test). (B–D) Scanning electron microscopy pictures of the abaxial epidermis of leaf 1 or 2 (21 DAS) from wild-type (B), *Promoter_bZIP29:bZIP29-SRDX* line 1 (C), or *Promoter_bZIP29:bZIP29-SRDX* line 2 (D). Scale bars: 100 μm. The insets show stomata of irregular (C) or normal (B and D) shape. (This figure is available in color at *JXB* online.)

To get a better understanding of how dominant-negative repression of *bZIP29* alters leaf development, a cellular analysis was performed on the abaxial leaf epidermis of the first leaf pair. In both *Promoter_bZIP29:bZIP29-SRDX* lines, there was a decrease in cell number, which was compensated by an increase in cell size (Fig. 4A). Because an increase in cell size often correlates with an increase in endoreduplication (Gonzalez *et al.*, 2012), ploidy levels of the first two leaves were analysed; however, no significant differences compared with wild-type were observed (Supplementary Fig. S5). Such an increase in cell size without increasing the amount of nuclear DNA has been shown before, involving either cell cycle genes (De Veylder *et al.*, 2001) or genes involved in cell wall modification (Cho and Cosgrove, 2000). Interestingly, in the line with the highest *bZIP29-SRDX* expression, asymmetric guard cells with irregular shapes were observed (Fig. 4C), in line with its expression in meristemoids and guard cells (Fig. 1G).

In contrast, ectopic overexpression of wild-type *bZIP29* from the 35S promoter led to impaired leaf growth with smaller leaves and a reduced number of leaves (Fig. 3D, *P35S:bZIP29*). The decrease in leaf area was the result of a decrease in both cell size and cell number (Fig. 4A). Taken together, there are converging lines of evidence that bZIP29 has an important function in leaf development through control of cell number or cell size.

### bZIP29 influences root gravitropic responses and root development

**Fig. 5.**
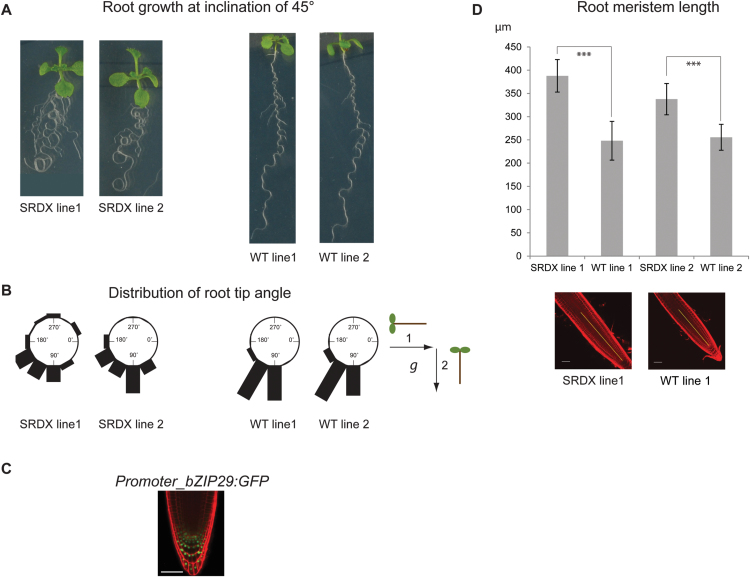
Phenotypical analysis of roots from *Promoter_bZIP29:bZIP29-SRDX* repressor lines. (A) Agravitropic root growth phenotype of two independent *Promoter_bZIP29:bZIP29-SRDX* repressor lines, compared with the wavy root growth phenotype of out-segregated wild-type plants, grown *in vitro* (7 DAS) at an inclination of approximately 45°. (B) Root gravitropic response of *Promoter_bZIP29:bZIP29-SRDX* repressor and wild-type lines following gravistimulation (as depicted in the scheme), determined by a root tip bending assay. (C) Confocal microscopy picture of the *Promoter_bZIP29:GFP-GUS* reporter line showing expression in the QC and columella cells. Roots were counterstained with propidium iodide. Scale bar: 50 µm. (D) Quantification of the root meristem size (µm) of *Promoter_bZIP29:bZIP29-SRDX* repressor and out-segregated wild-type lines. ***P<0.001 (Student’s *t*-test). Representative pictures of root tips from repressor and wild-type lines, counterstained with propidium iodide, are shown below the graph. Length of meristem is shown by yellow lines. Scale bars: 50 µm. (This figure is available in color at *JXB* online.)

Because *bZIP29* is expressed in the meristem of the primary root and in the lateral root primordia (Fig. 1), the roots of the two dominant-negative repressor lines were phenotypically screened. Although no differences in primary root length and lateral root number were observed (Supplementary Fig. S6A), their roots clearly showed a wavy root phenotype, pointing to a disturbance of the gravitropic response. When plants were grown on inclined plates at an angle of 45°, roots started to loop with more severe effects on gravitropic responses (Fig. 5A). The impaired gravitropic response was confirmed and quantified in a root tip bending assay (Fig. 5B). The altered gravitropic response was in line with *bZIP29* expression in the gravity-perceiving columella cells of the root tip, as seen by GUS staining (Fig. 1B) and by confocal analysis of the *Promoter_bZIP29:GFP-GUS* reporter line (Fig. 5C). However, no alterations in amyloplast distribution were observed in the columella cells (Supplementary Fig. S6B). Recently, a similar root waving phenotype was observed in a VIP1-SRDX overexpressor line (Tsugama *et al.*, 2016), which was linked to changes in local auxin responses. In addition, roots of both *Promoter_bZIP29:bZIP29-SRDX* lines showed an increase in root meristem size (Fig. 5D). In line with the increased root meristem size, 4′,6-diamidino-2-phenylindole (DAPI) staining of root tips confirmed an increase in the number of meristematic nuclei (Supplementary Fig. S6C). Because bZIP29 is mainly expressed in the QC of the root tip, the *Promoter_bZIP29:bZIP29-SRDX* repressor line 1 was crossed with a *Promoter_QC25:CFP* (cyan fluorescent protein) marker line; however, no abberrant QC divisions or QC organization were observed (Supplementary Fig. S6D).

**Fig. 6.**
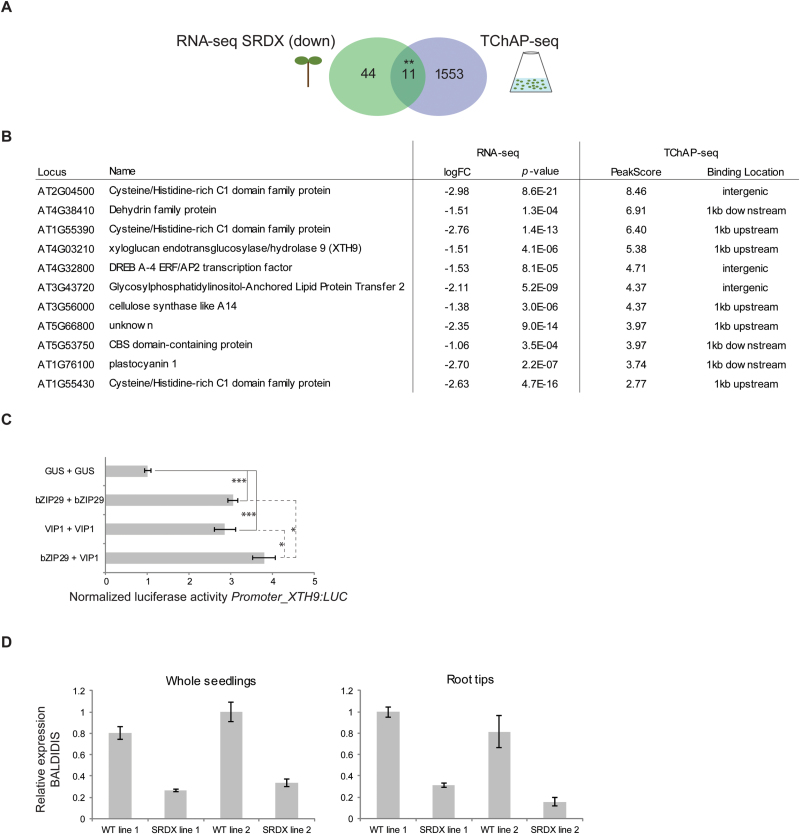
Transcriptome analysis of root tips from the *Promoter_bZIP29:bZIP29-SRDX* repressor line 1 and comparison with the TChAP-seq target list. (A) Venn diagram showing a significant overlap (**P=3.87×10^–5^) between genes bound by bZIP29 identified by TChAP-seq in cell culture, and genes down-regulated in the root tips of the *Promoter_bZIP29:bZIP29-SRDX* line 1 as identified by RNA-seq. (B) Summary of 11 direct target genes bound by bZIP29 and down-regulated upon repression of *bZIP29* in root tips. (C) bZIP29- and/or VIP1-dependent activation of the *XTH9* promoter in a transient expression assay. Different combinations of bZIP29 and VIP1 were tested. Equal total amount of effector plasmids was used. Indicated values represent average relative luciferase activities compared with a *P35S:GUS* control for eight biological repeats. ***P<0.001; *P<0.05 (Student’s *t*-test). (D) Relative expression of *BALDIBIS* (AT3G45260) in seedlings and in root tips from the *Promoter_bZIP29:bZIP29-SRDX* repressor lines and the out-segregated wild-types, as determined by qRT-PCR. Expression was normalized using *Actine2_2* and *AteEF1A-4* as reference genes. (This figure is available in color at *JXB* online.)

To investigate the mechanism by which bZIP29 controls root development, a transcriptome analysis was performed to screen for genes that are differentially expressed in the root tips of the repressor line with the highest *bZIP29-SRDX* expression. Compared with the out-segregated wild-type, 171 genes (Supplementary Table S5) showed an altered expression profile, of which 55 genes were repressed. According to a gene ontology enrichment analysis (Thimm *et al.*, 2004), ‘cell wall’ (*P*=6.48×10^–7^) was significantly enriched among the 171 differentially expressed genes, in agreement with the gene ontology enrichment analysis on the TChAP-seq data. When the set of down-regulated genes was compared with that of the TChAP-seq data, we identified a small but significant overlap (*P*=3.87×10^–5^) of 11 genes that were repressed in the transgenic root tips (Fig. 6A, B), while there was only one gene from the TChAP-seq target list that overlapped with the 116 up-regulated genes. Thus almost all genes bound by bZIP29 and differentially expressed in the root meristem are repressed. This was expected because fusion of the SRDX domain leads to dominant-negative repression, and therefore direct target genes should be down-regulated. Among these 11 genes, two are involved in cell wall organization: a cellulose synthase like gene (*CSLA14*) and the *XTH9* gene discussed before. Direct activation of *XTH9* by bZIP29 was confirmed in a transient assay with the *XTH9* promoter driving expression of a luciferase reporter gene (Fig. 6C). Interestingly, activation of the *XTH9* promoter was stronger when bZIP29 was combined with VIP1, providing evidence that bZIP group I members synergistically activate gene transcription as heterodimers, and indicating that bZIP29 might act together with other interacting bZIP group I factors to control root development. One of the strongest down-regulated genes in the transcriptome data (log2(fold change)=–3.12) was *BALDIBIS*, encoding a TF from the plant-specific C2H2 zinc finger family. Together with its homolog, JACKDAW, BALDIBIS regulates tissue boundaries and asymmetric cell division in the root meristem through control of SHORT-ROOT and SCARECROW activity (Welch *et al.*, 2007; Ogasawara *et al.*, 2011; Long *et al.*, 2015). The down-regulation of *BALDIBIS* was confirmed in both *Promoter_bZIP29:bZIP29-SRDX* lines, both in root tips and in seedlings (Fig. 6D). In the RNA-seq data (Supplementary Table S5), many additional TFs were present. Notably, the list contains seven members of the AGAMOUS-like MADS-box TF family, of which all but one (AGL87) belong to the MIKC-type clade. Although many members of this subfamily are well known for their role in flower development, some members are required for proper root development (Nawy *et al.*, 2005; Tapia-López *et al.*, 2008; Moreno-Risueno *et al.*, 2010; Hacham *et al.*, 2011). The root tip transcriptome analysis shows thus that expression of cell wall regulators is altered and that gene regulatory networks in the root are intensively rewired upon perturbation of bZIP29.

## Discussion

To characterize the function of bZIP29 during plant development, we analysed its expression pattern, demonstrating *bZIP29* expression in proliferative tissues and not in vascular tissues. This supports its previous identification in a cell cycle interactome (Van Leene *et al.*, 2010) and suggests a function in proliferating cells. Moreover, transcriptome data indicate upstream regulation of *bZIP29* expression by auxin in different root cell types (Bargmann *et al.*, 2013). This correlates with the observed expression pattern of *bZIP29* in the QC and columella cells, where auxin levels peak. Auxin-dependent expression of *bZIP29* is further supported by the presence of an auxin-response element (TGTCTC) in its promoter, 295bp upstream of the translation start. In a recent phosphoproteomics study (Zhang *et al.*, 2013), bZIP29 has been identified to be phosphorylated during auxin-mediated lateral root initiation, supporting its expression in the lateral root primordia. Also for stomata, transcriptome and proteome profiling studies have validated *bZIP29* expression in this cell type (Zhao *et al.*, 2008; Obulareddy *et al.*, 2013) and transcriptome data indicate that expression of *bZIP29* in guard cells is down-regulated by ABA (Pandey *et al.*, 2010).

To get more insight into the downstream target genes that are regulated by bZIP29, we performed TChAP-seq analyses. In agreement with partially overlapping functions of bZIP29 and VIP1 (Tsugama *et al.*, 2014), *de novo* motif analysis identified a motif resembling the VIP1-responsive element involved in stress signaling and the hypo-osmolarity-response element, and all known targets of VIP1 were present in the bZIP29 target list. Moreover, this motif is similar to motifs linked to the bZIP group I factors bZIP30 and bZIP18, demonstrating the functional relevance of the TChAP-seq data. Previously, it has been reported that two key enzymes in ABA degradation show specific expression patterns, with *CYP707A3* expressed in vascular tissue and *CYP707A1* in stomata (Okamoto *et al.*, 2009). Integrating earlier reports on VIP1 with our TChAP-seq and expression data of *bZIP29*, we propose a model in which *CYP707A3* is activated by VIP1 in vascular tissue and *CYP707A1* by bZIP29 in the stomata. The motif was, however, not present in all peaks, indicating a more complex DNA-binding specificity and additional roles for bZIP29.

The gene ontology enrichment analysis on the target gene list further indicated that bZIP29 controls expression of a broad range of downstream targets, pointing to a pleiotropic role for this TF, similar to the multifunctionality of VIP1. In agreement with its expression profile in proliferative tissues and with its previous identification in the cell cycle interactome, we identified an over-representation of bZIP29 target genes that are periodically expressed during the cell cycle. Also many links with the cell wall were found, suggesting that bZIP29 might be an important player in growth through control of cell wall organization during cell proliferation. Moreover, there is an increasing amount of evidence that implies a general role for bZIP group I members in cell wall organization: tomato VSF-1 regulates expression of a cell wall protein in vascular tissue (Ringli and Keller, 1998), tobacco RSG regulates cell expansion in phloem cells (Fukazawa *et al.*, 2000), and upon rehydration, up-regulated genes containing the VIP1-responsive element are mainly linked to cell wall organization (Tsugama *et al.*, 2014).

**Fig. 7.**
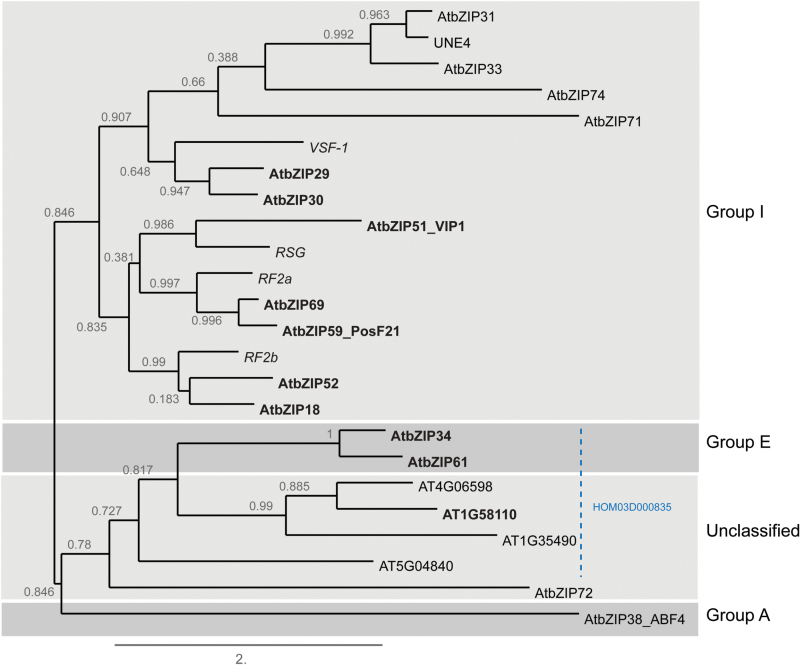
Rooted maximum likelihood phylogenetic tree encompassing all Arabidopsis bZIP group I and group E members, and their bZIP interactors as identified by TAP. In addition, four unclassified bZIP factors from Arabidopsis are included, forming one subfamily (HOM03D000835) (dashed line) with the two group E members, according to the PLAZA 3.0 Dicots comparative genomics platform. PLAZA also clustered the unclassified AtbZIP72 (AT5G07160) as part of the same subfamily (HOM03D000341) as the group I members. AtbZIP73 (AT2G13130) was excluded from the analysis, since it encodes a pseudogene, while UNE4 (AT2G12940) was added to group I because it contains the conserved lysine residue and is part of the HOM03D000341 subfamily. Proteins identified by TAP with bZIP29 are shown in bold. Group I orthologs from other plant species are shown in italics. The group A member ABF4/AtbZIP38 was used as an out-group. Protein sequences from Arabidopsis were based on the TAIR10 annotation (Lamesch *et al.*, 2012) and orthologous protein sequences were obtained from UNIPROT (Dimmer *et al.*, 2012). The PhyML tree was constructed using the ‘one click’ mode of the Phylogeny.fr software tool (Dereeper *et al.*, 2008). The branch length is proportional to the number of substitutions per site and branches are annotated with their support values. (This figure is available in color at *JXB* online.)

To elucidate the protein complexes in which bZIP29 resides, we performed a protein–protein interaction screen. This analysis confirmed that bZIP29 interacts with multiple group I members. A closer look at the group I proteins showed that they form only heterodimers within a phylogenetic subgroup of seven proteins, to which also the orthologous RSG, VSF-1, RF2a and RF2b proteins cluster (Fig. 7). The heterodimerization of bZIP29 with other bZIP factors is not restricted to members of group I. bZIP29 also interacts with closely related group E members and with one unclassified bZIP TF. The unclassified bZIP TF forms one subfamily (HOM03D000835) with two other unclassified bZIP TFs and with both group E members according to PLAZA and our phylogenetic analysis (Fig. 7). The unclassified members, however, do not contain the proline residue present in group E members nor the lysine residue present in group I members (Supplementary Fig. S3). Homo- and heterodimerization of bZIP TFs will result in unique pairing of DNA-binding preferences and transactivation domains, giving rise to bZIP dimers with unique functions (Schütze *et al.*, 2008). Moreover, heterodimerization might lead to stronger activation compared with homodimerization, as we demonstrated for *XTH9*. A recent study shows that the dimerization preference of bZIP TFs (group C member bZIP63) might be regulated through phosphorylation (Mair *et al.*, 2015). Putative kinases that could phosphorylate bZIP29 are the mitotic CDKB–cyclin B complexes, identified previously as interactors of bZIP29 (Van Leene *et al.*, 2010), and the mitogen-activated protein kinase MPK3 (Pitzschke *et al.*, 2009), which phosphorylates and activates VIP1 during the stress response. Furthermore, phosphorylation might lead to the observed interactions with 14-3-3 GRFs. Binding to 14-3-3 proteins can have both positive and negative effects, altering the subcellular localization, the protein stability or the interaction partners of the bound protein (Oecking and Jaspert, 2009). Regulation of bZIP group I members by 14-3-3 proteins has been demonstrated before. Phosphorylation of tobacco RSG leads to association with 14-3-3 proteins and sequestration in the cytosol (Igarashi *et al.*, 2001). Similar to the regulation of RSG, phosphorylation might alter the localization of bZIP29 through interaction with 14-3-3 proteins. Interestingly, the serine residue (S196) in one of the 14-3-3 motifs of bZIP29 has been identified to be phosphorylated in two phosphoproteome studies related to ABA signaling or dehydration (Umezawa *et al.*, 2013; Wang *et al.*, 2013). Because bZIP29 regulates expression of ABA-degrading enzymes, these data suggest that phosphorylation and interaction with 14-3-3 proteins might act negatively on bZIP29. An extra level of regulation of bZIP group I members might be achieved through interaction with a PP2A phosphatase complex. Such a phosphatase complex might counteract phosphorylation and binding to 14-3-3 proteins. In concert with this hypothesis, PP2A complexes have been shown before to act negatively in ABA responses (Pernas *et al.*, 2007). When screening transcriptome data using the Arabidopsis eFP browser (Winter *et al.*, 2007), expression of *ATB′′β* and *ATB′′δ* was down-regulated in stomata upon ABA treatment (Supplementary Fig. S7). Taken together, this suggests a mechanism in stomata or vascular tissues where upon rehydration, PP2A complexes dephosphorylate respectively bZIP29 or VIP1, counteracting their ABA- or dehydration-induced phosphorylation, binding by 14-3-3 proteins and sequestration in the cytosol. As a consequence, bZIP29 and VIP1 might translocate to the nucleus, where they activate gene expression of *CYP707A1* in stomata or *CYP707A3* in vascular tissue.

In addition to a function of bZIP29 and redundant factors in stress signaling or osmosensory response, the expression analysis and the TChAP data indicated that bZIP29 might have additional functions during plant development. Constitutive dominant-negative repression of *bZIP29* was seedling-lethal, implying an essential role for bZIP29 and redundant factors early during plant development. In contrast, constitutive dominant-negative repression of VIP1 is not seedling-lethal (Tsugama *et al.*, 2016), pointing to a partial functional divergence between bZIP29 and VIP1. Dominant-negative repression controlled by its endogenous promoter revealed more specific functions of bZIP29, leading to an explicit leaf serration phenotype and a reduction in cell number. These observations further support a role for bZIP29 in cell proliferation, because a higher degree of leaf serration is a general indication of misregulated cell divisions (De Veylder *et al.*, 2001; Wu *et al.*, 2005). The decrease in cell number is compensated by an increase in cell size, which is not linked to an increase in ploidy levels, restoring the final leaf size or even generating larger leaves. In previous reports, however, this compensation effect almost never restores the final leaf size. Overproduction of the CDK inhibitor KIP-related protein 2 (KRP2), for example, inhibits cell cycle progression, giving rise to more serrated leaves and enlarged cells, but the final leaf size was smaller compared with wild-type (De Veylder *et al.*, 2001; Verkest *et al.*, 2005; Blomme *et al.*, 2014). The leaf size-controlling compensation mechanism is typical for mutants with altered cell cycle gene expression and is often associated with an enhanced onset of differentiation (Blomme *et al.*, 2014). This could point to a role for bZIP29 in suppression of differentiation through regulation of cell cycle genes or genes involved in cell wall organization.

In concert with the presence of bZIP29 in the root apical meristem and in the gravity-perceiving columella cells, developmental defects were also observed in the roots of the repressor lines. The roots showed a wavy growth pattern, which is often an indication of a disturbed gravitropic response. Interestingly, the root meristem size of both transgenic lines was increased, which might be a consequence of the perturbation of bZIP29 activity in the QC, because the latter orchestrates proper root meristem organization (Sozzani and Iyer-Pascuzzi, 2014). Remarkably, the increase in root meristem size upon *bZIP29* repression is the opposite of the reduction in cell number observed in leaves. Such opposing effects in leaves and roots have been reported before: cytokinine phytohormones, for example, have central, but opposite, regulatory functions in root and shoot meristems (Werner *et al.*, 2003).

The transcriptome analysis on the dominant-negative repression line confirmed that bZIP29 regulates genes involved in cell wall organization. Furthermore, bZIP29 controls a complex gene regulatory network in the root, because expression of many TFs was perturbed in the root tips of the dominant-negative repression line.

**Fig. 8.**
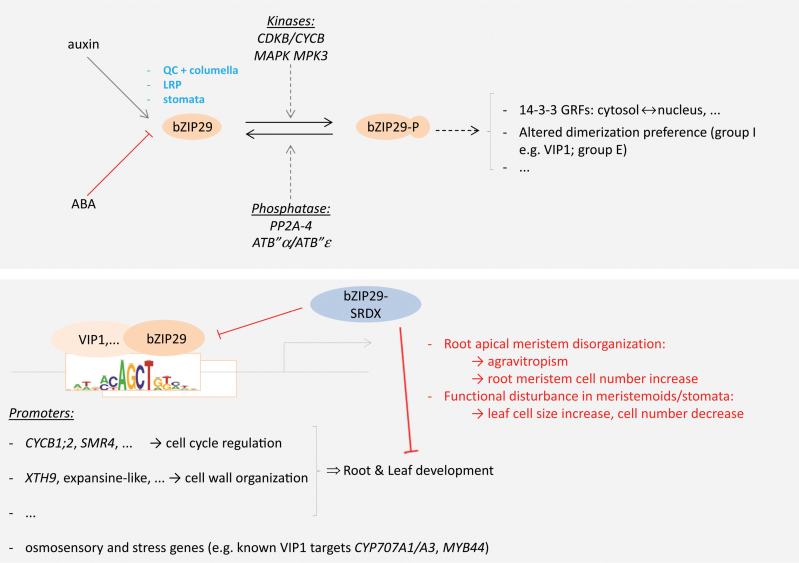
Summarizing model on bZIP29 function and regulation. bZIP29 is mainly expressed in the QC, columella cells, lateral root primordia (LRP), meristemoids, and stomata. Upstream, bZIP29 is transcriptionally regulated by auxin (+) or ABA (–). Post-translational regulation of bZIP29 activity might be obtained through activating or inhibiting phosphorylation by mitotic CDKB–cyclin B kinase complexes or MPK3, and counteracted by PP2A-4-containing phosphatase complexes. Phosphorylation could regulate its subcellular localization through interaction with 14-3-3 GRFs, or its dimerization preference with other bZIP TFs like VIP1. Homo- or heterodimers of bZIP29 regulate expression of osmosensory and stress genes, and genes involved in cell cycle regulation or cell wall organization, through binding to the core motif mCAGCTGk or other *cis*-regulatory elements. Repression of bZIP29 and redundant factors alters leaf and root development. (This figure is available in color at *JXB* online.)

This study broadens the knowledge of bZIP group I members, showing that this subfamily is essential for plant development, and revealing possible means of their post-translational regulation. This knowledge is projected in a model (Fig. 8), although further studies are needed to unravel the causality between the regulation of cell cycle or cell wall genes by bZIP29 and the control of leaf and root development.

## Supplementary data

Supplementary data are available at *JXB* online.

Figure S1. bZIP29 TChAP-seq results of known VIP1 targets.

Figure S2. GUS staining of a *Promoter_bZIP69:GFPGUS* reporter line.

Figure S3. Multiple alignment of plant bZIP group I, group E and related bZIP factors used to construct the phylogenetic tree (Fig. 7).

Figure S4. Relative expression level (*n*=3) of the *bZIP29-SRDX* transcript.

Figure S5. DNA ploidy analysis of leaf 1 and 2.

Figure S6. Primary root length and lateral root density analysis, lugol staining of amyloplasts, DAPI staining, and QC25-CFP marker expression analysis.

Figure S7. Expression analysis of protein phosphatases 2A (PP2A) regulatory B′′ subunits, with or without ABA treatment.

Table S1. List of primers used for cloning, T-DNA insertion or qPCR analyses.

Table S2. Protein identification details obtained with the LTQ Orbitrap Velos (Thermo

Fisher Scientific) on the bZIP29 seedling TAPs (Table S2A), or with the 4800 MALDI TOF/TOF Proteomics analyzer (AB SCIEX) on the bZIP29 cell culture TAPs (Table S2B).

Table S3. TChAP intersection list of 1678 annotated peaks found in both replicates overlapping for at least 25% (Table S3A), and individual TChAP results of replicate 1 (Table S3B) and replicate 2 (Table S3C).

Table S4. Gene ontology classes (biological process, molecular function, cellular compartment) that are enriched in the list of genes bound by bZIP29 (intersection of both replicates), as determined by the gene ontology enrichment tool integrated in the PLAZA comparative genomics platform.

Table S5. List of genes differentially expressed (twofold; corrected *P*-value <0.05; FDR <0.05) in root meristems of *Promoter_bZIP29:bZIP29-SRDX* line 1 (compared with the out-segregated WT line 1) identified by RNA-seq transcriptome analysis.

Supplementary Data

## Abbreviations:

ABA: abscisic acid
BiFC: bimolecular fluorescence complementation
bZIP: basic region/leucine zipper
CDS: coding sequence
GRF: general regulatory factor
MS: Murashige and Skoog
TAP: tandem affinity purification
TChAP: tandem chromatin affinity purification
TF: transcriptions factor
QC: quiescent center
VIP1: VirE2-interacting protein 1
VRE: VIP1 response element
Y2H: yeast two-hybrid.
