# Supplementary material for: Functional characterization of the Arabidopsis transcription factor bZIP29 reveals its role in leaf and root development
Source: J Exp Bot. 2016 Sep 22;67(19):5825–40. doi: 10.1093/jxb/erw347 (PMC5066499; doi:10.1093/jxb/erw347)
Supplement: Supplementary Data [file supp_67_19_5825__index.html]

Functional characterization of the Arabidopsis transcription factor bZIP29 reveals its role in leaf and root development — Functional characterization of the Arabidopsis transcription factor bZIP29 reveals its role in leaf and root development — Supplementary Data 

# Functional characterization of the Arabidopsis transcription factor bZIP29 reveals its role in leaf and root development

## Supplementary Data

Data files

- Supplementary\_Tables\_S1\_and\_Figures\_S1\_S7.pdf - Supplementary Data
- Supplementary\_Tables\_S2A\_and\_2B.xls - Supplementary Data
- Supplementary\_Table\_S3.xls - Supplementary Data
- Supplementary\_Table\_S4.xls - Supplementary Data
- Supplementary\_Table\_S5.xls - Supplementary Data
