# Supplementary material for: Functional characterization of the Arabidopsis transcription factor bZIP29 reveals its role in leaf and root development
Source: J Exp Bot. 2016 Sep 22;67(19):5825–40. doi: 10.1093/jxb/erw347 (PMC5066499; doi:10.1093/jxb/erw347)
Supplement: Supplementary Data [file supp_erw347_Supplementary_Tables_S1_and_Figures_S1_S7.pdf]

SUPPLEMENTAL TABLES

Table S1. List of primers used for cloning, T-DNA insertion or qPCR analyses.

Class	Oligo name	Sequence
cloning bait	attB1short-bZIP29_fwd	AAAAAGCAGGCTCCACCATGGGTGATACAGAGA AGTGTAACAGTG
cloning bait	attB2short-bZIP29_closed_rev	AGAAAGCTGGGTCCTATTCATTTGATTCAGATTTT GTTGCC
cloning bait	attB2short-bZIP29_open_rev	AGAAAGCTGGGTCTTCATTTGATTCAGATTTTGTT GCC
cloning SRDX	attB2R-SRDX-bZIP29_rev	GGGGACCACTTTGTACAAGAAAGCTGGGTGTCA AGCGAAACCCAAACGGAGTTCTAGATCCAGATCG AGTTCATTTGATTCAGATTTTGTTG
cloning promoter	attB4-ProbZIP29_fwd	GGGGACAACTTTGTATAGAAAAGTTGTAGATCGG TAGATCATATCCGTTGCA
cloning promoter	attB1R-ProbZIP29_rev	GGGGACTGCTTTTTTGTACAAACTTGTTTAGATC GGATAATGCAGTTTCTAATTCT
cloning promoter	attB4-ProbZIP69_fwd	GGGGACAACTTTGTATAGAAAAGTTGCAGTCAAT CTTGGTTACCATTTTGACAT
cloning promoter	attB1R-ProbZIP69_rev	GGGGACTGCTTTTTTGTACAAACTTGTTCAAGAA CTTGACCTAAACCCACTAATC
cloning promoter	attB1_Pro_XTH9	GGGGACAAGTTTGTACAAAAAAGCAGGCTCCAG AGTCTAATGAAAAGTGAGTGAGA
cloning promoter	attB2_Pro_XTH9	GGGGACCACTTTGTACAAGAAAGCTGGGTCTTTT TTTTTAACTTATCTCTCTAAATAAATCAAACTTC
qPCR	qPCR_bZIP29_fwd	ACGCTGAGATGTTCCAGCAA
qPCR	qPCR_SRDX_rev	CAAGCGAAACCCAAACGGAG
qPCR	PP2A_fwd	TAACGTGGCCAAAATGATGC
qPCR	PP2A_rev	GTTCTCCACAACCGCTTGGT
qPCR	ACT2_2_FWD	TTGACTACGAGCAGGAGATGG
qPCR	ACT2_2_REV	ACAAACGAGGGCTGGAACAAG
qPCR	EEF1A4_FWD	CCAAGGGTGAAAGCAAGAAGA
qPCR	EEF1A4_REV	CTGGAAGGTTTTGAGGCTGGTAT
qPCR	BALDIBIS_fwd	CCCTAACCCTAATCCTACCTCCT
qPCR	BALDIBIS_rev	CGATGACTTCTGCATCTGGA
T-DNA confirmation	LP_GABI_211B01	AGGCGGCTTTAGAGATTCATC
T-DNA confirmation	RP_GABI_211B01	CAAAGCTTGATCTTTTAGGGG

Table S2. Protein identification details obtained with the LTQ Orbitrap Velos (Thermo Fisher Scientific) on the bZIP29 seedling TAPs (Table S2A), or with the 4800 MALDI TOF/TOF Proteomics analyzer (AB SCIEX) on the bZIP29 cell culture TAPs (Table S2B).

See Supplementary Table S2.xls with two sheets.

Table S3. TChAP intersection list of 1678 annotated peaks found in both replicates overlapping for at least 25% (Table S3A), and individual TChAP results of replicate 1 (Table S3B) and replicate 2 (Table S3C).

See Supplementary Table S3.xls with three sheets.

Table S4. Gene ontology classes (Biological Process, Molecular Function, Cellular Compartment) that are enriched in the list of genes bound by bZIP29 (intersection of both replicates), as determined by the gene ontology enrichment tool integrated in the PLAZA comparative genomics platform.

See Supplementary Table S4.xls.

Table S5. List of genes differentially expressed (twofold; corrected *p*-value <0.05; FDR <0.05) in root meristems of *Promoter_bZIP29:bZIP29-SRDX* line 1 (compared with the out-segregated WT line 1) identified by RNA-seq transcriptome analysis.

See Supplementary Table S5.xls.

Supplemental Figure S1. bZIP29 TChAP-seq results of known VIP1 targets

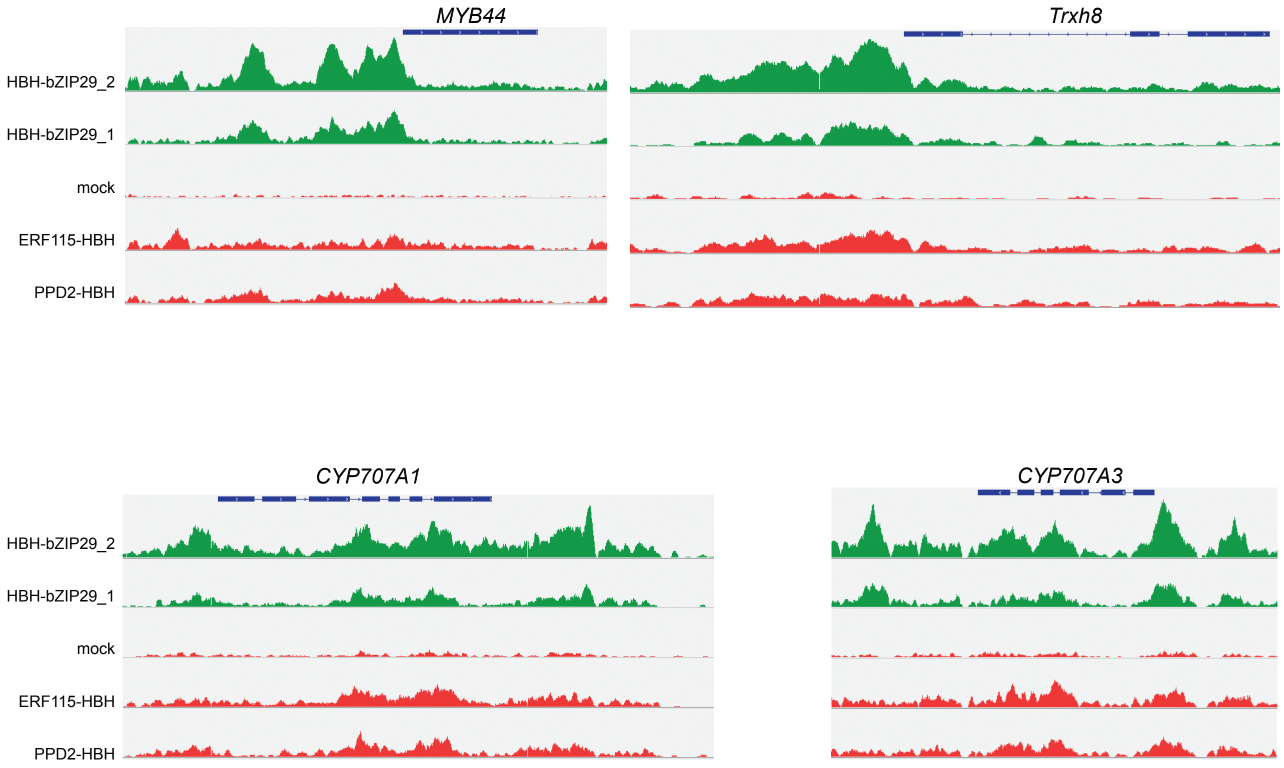

IGV screenshot of sequenced reads from bZIP29, mock, ERF115, and PPD2 TChAP-seq experiments, mapped to all four known bZIP51/VIP1 targets *MYB44* (AT5G67300), *Trxh8* (AT1G69880), *CYP707A1* (AT4G19230), *CYP707A3* (AT5G45340). The gene regions are represented by blue bars.

Supplemental Figure S2. Histochemical GUS staining of a *Promoter_bZIP69:GFPGUS* reporter line.

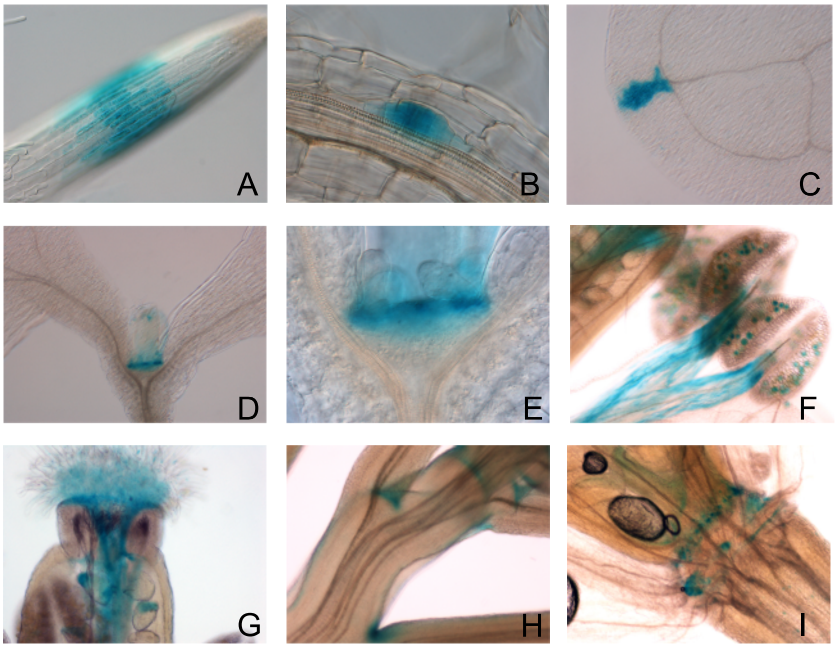

(A-I) Histochemical GUS staining of a *Promoter_bZIP69:GFPGUS* reporter line. Expression was detected in the lateral root cap of the primary root (A) and in the lateral root primordia (B). In leaves, GUS staining was observed in the hydathode pores (C), structures related to stomata, and in the shoot apical meristem (D & E).
During flower development, expression was detected in anther filaments and pollen (F), and in the style and stigma (G). Furthermore, *bZIP69* was expressed at pedicel-stem junctions (H) and at the abscission zones of sepals and petals (I).

Supplemental Figure S3

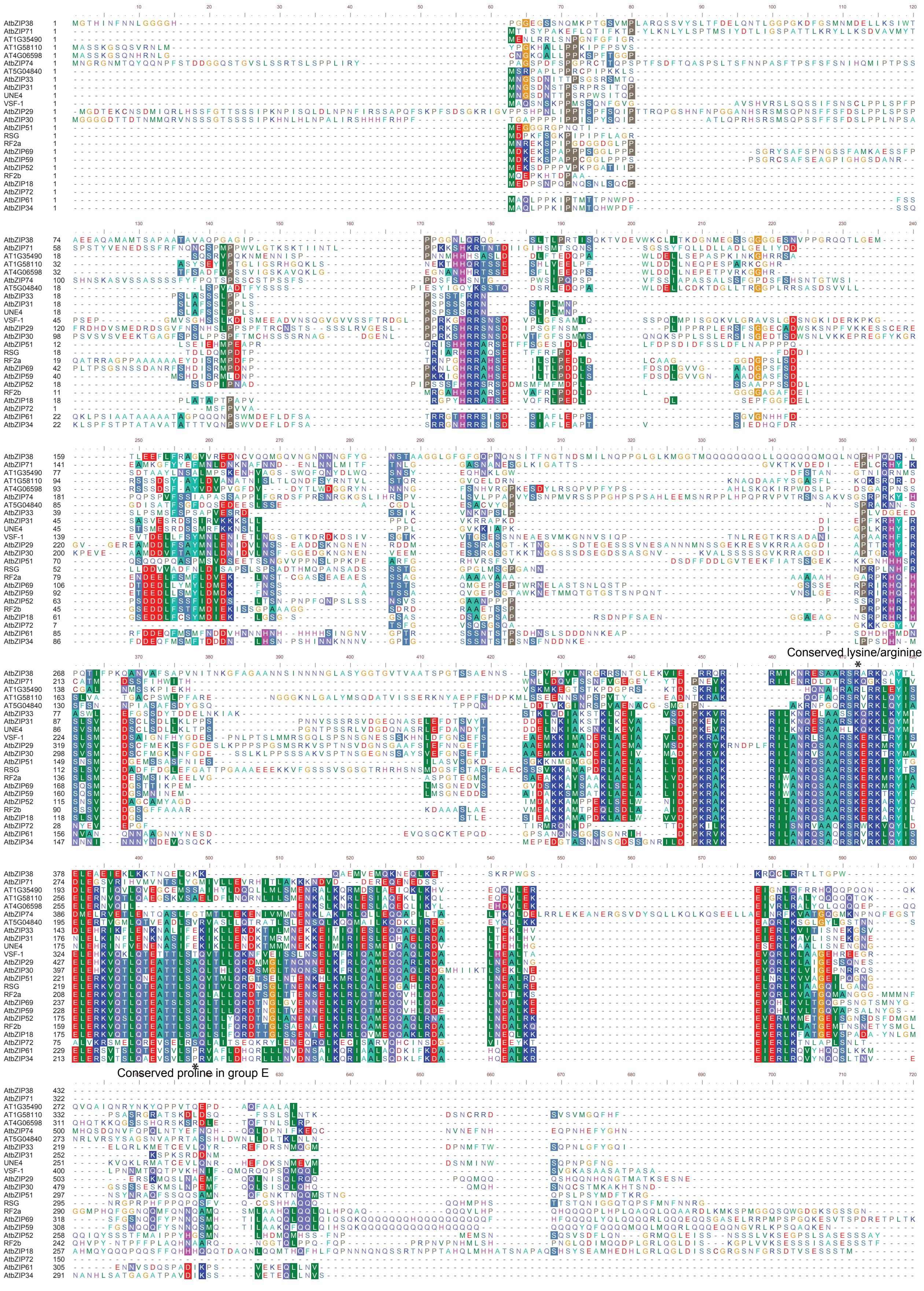

Multiple alignment of plant bZIP group I, group E and related bZIP factors used to construct the phylogenetic tree (Figure 7). The conserved lysine residue in the basic domain that replaces the highly conserved arginine residue in group I members, and the conserved proline residue preventing homodimerization of group E members are shown. Identical and similar residues are shaded based on the color table from BioEdit. (http://www.mbio.ncsu.edu/bioedit/bioedit.html)

Supplemental Figure S4

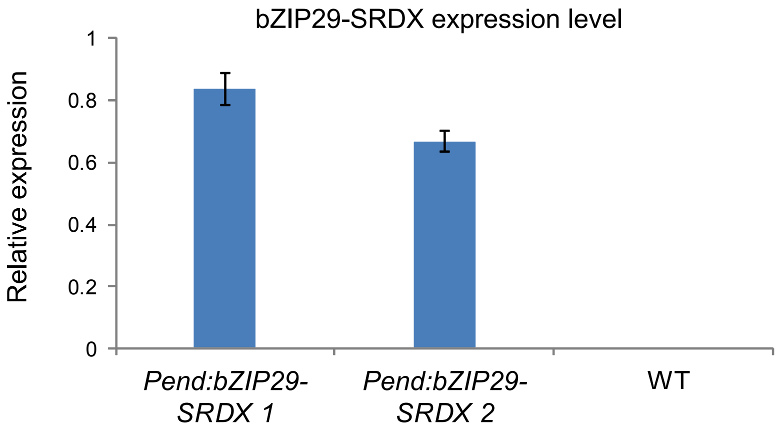

Relative expression level (n=3) of the *bZIP29-SRDX* chimeric transcript, extracted from two independent *Promoter_bZIP29:bZIP29-SRDX* lines, as determined by qRT-PCR, normalized against the *PP2A* reference gene.

Supplemental Figure S5. DNA ploidy analysis of leaf 1 and 2.

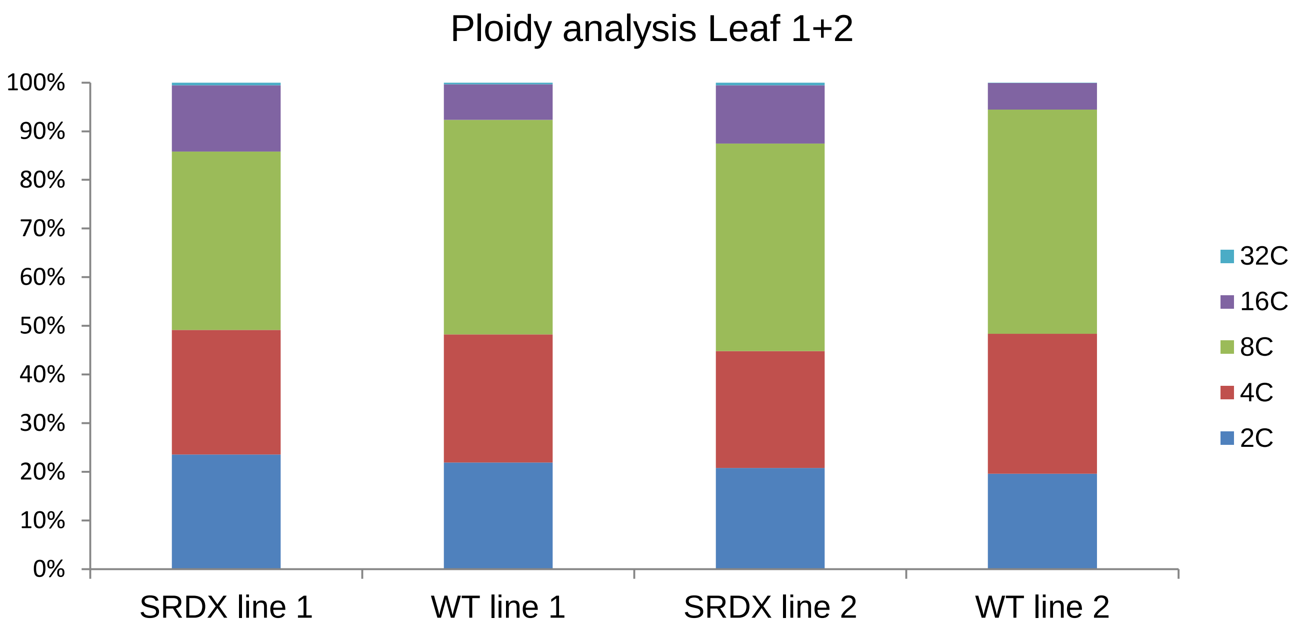

DNA ploidy analysis of leaf 1 and 2, 21 DAS, of both *Promoter_bZIP29:bZIP29-SRDX* and out-segregated wild-type lines, determined by flow cytometry analysis as described in Blomme *et al*. 2014.

Supplemental Figure S6

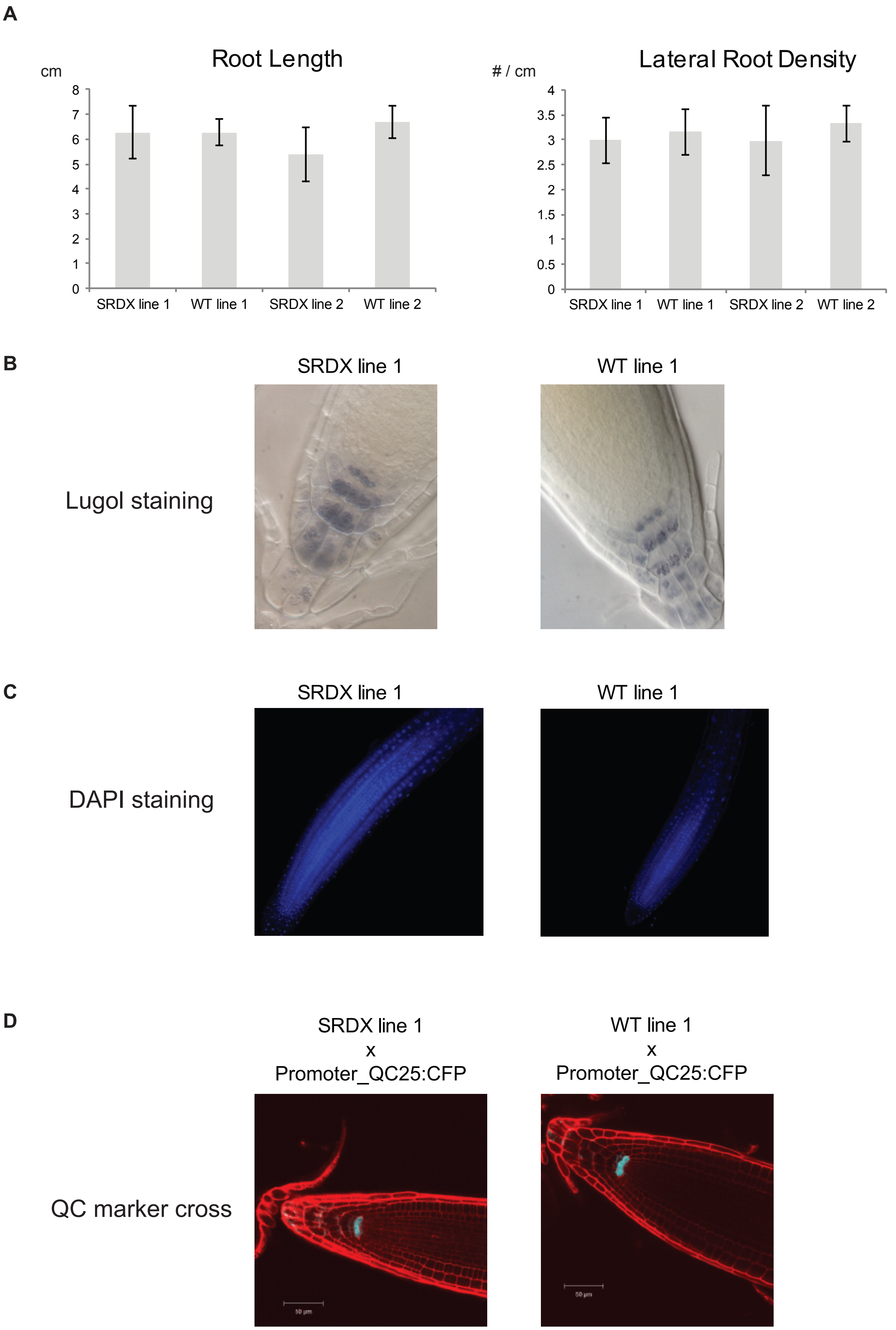

(A) Primary root length and lateral root density (# lateral roots/ primary root length) of both *Promoter_bZIP29:bZIP29-SRDX* and out-segregated wild-type lines, 10 DAS. Error bars represent the standard deviation, *n* ≥ 19. (B) Lugol staining of amyloplasts in columella cells of root tips from *Promoter_bZIP29:bZIP29-SRDX* line 1 and out-segregated wild-type line 1, 7 DAS. (C) Confocal analysis of DAPI stained root tips, 5 DAS, showing increased meristematic zone of the *Promoter_bZIP29-SRDX* line 1 compared to wild type. DAPI staining was performed as described (Ishida et al, 2009, Plant Cell, SUMO E3 ligase HIGH PLOIDY2 regulates endocycle onset and meristem maintenance in Arabidopsis). (D) Confocal analysis after propidium iodide staining of root tips of *Promoter_bZIP29:bZIP29-SRDX* line 1 and out-segregated wild-type line 1, crossed with a *Promoter_QC25:CFP* QC marker line (Blilou et al, Nature 2005).

Supplemental Figure S7

AT5G28850: ATB”ε AT5G28900: ATB”δ

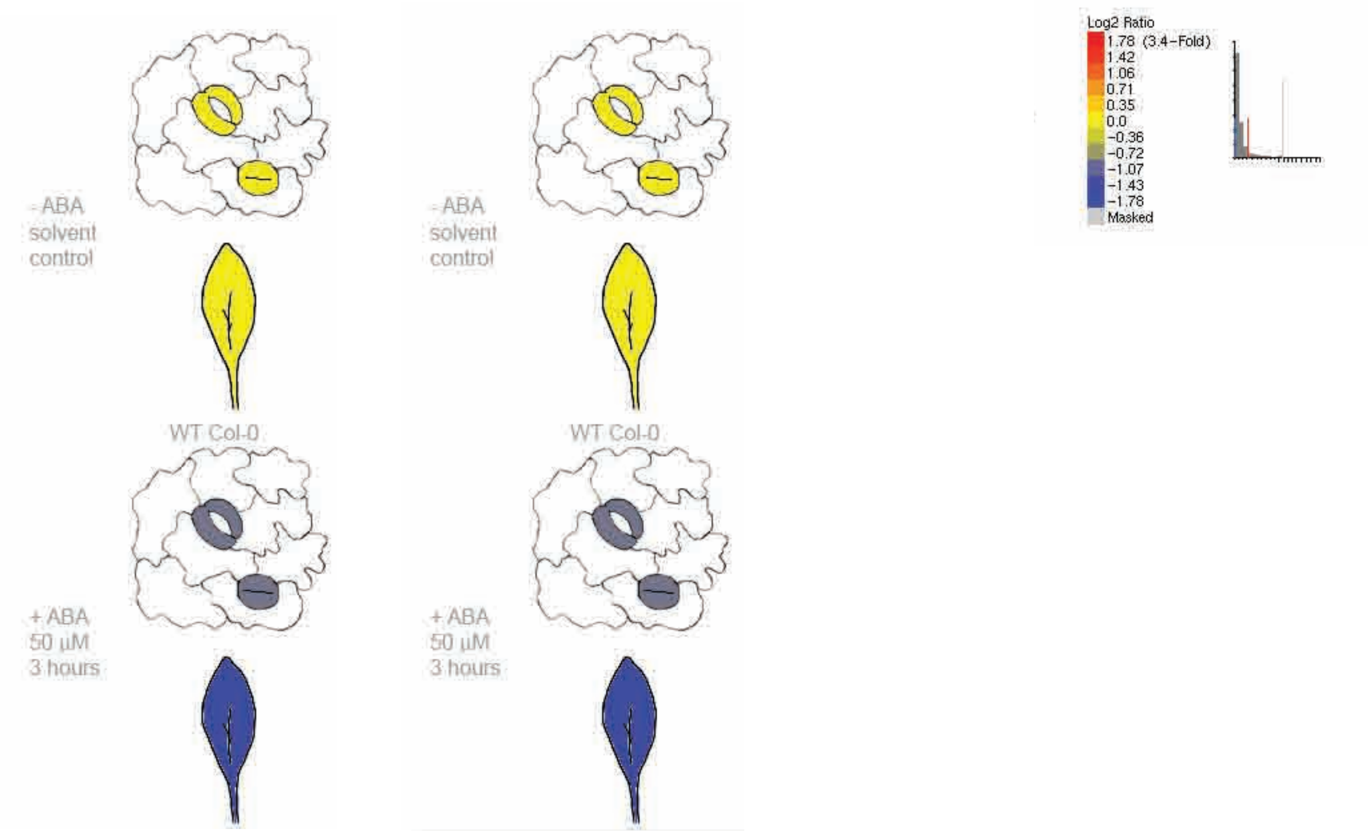

Expression analysis of protein phosphatases 2A (PP2A) regulatory B” subunits *ATB”ε* and *ATB”δ* in Col-0 wild-type stomata and leaves, with or without ABA treatment. Relative expression values are visualized using the Arabidopsis eFP browser.
